# Signal Processing for the Condition-Based Maintenance of Rotating Machines via Vibration Analysis: A Tutorial

**DOI:** 10.3390/s24020454

**Published:** 2024-01-11

**Authors:** Omri Matania, Lior Bachar, Eric Bechhoefer, Jacob Bortman

**Affiliations:** 1 BGU-PHM Laboratory, Department of Mechanical Engineering, Ben-Gurion University of the Negev, P.O. Box 653, Beer Sheva 8410501, Israel; liorbac@post.bgu.ac.il (L.B.); jacbort@bgu.ac.il (J.B.); 2 GPMS International Inc., 93 Pilgrim Place, Waterbury, VT 05676, USA; eric@gpms-vt.com

**Keywords:** condition-based maintenance, signal processing, vibration, rotating machines, tutorial

## Abstract

One of the common methods for implementing the condition-based maintenance of rotating machinery is vibration analysis. This tutorial describes some of the important signal processing methods existing in the field, which are based on a profound understanding of the component’s physical behavior. Furthermore, this tutorial provides Python and MATLAB code examples to demonstrate these methods alongside explanatory videos. The goal of this article is to serve as a practical tutorial, enabling interested individuals with a background in signal processing to quickly learn the important principles of condition-based maintenance of rotating machinery using vibration analysis.

## 1. Introduction

Complex mechanical systems such as helicopters, trains, and wind turbines require expensive maintenance to prevent accidents that can cost human lives or cause severe damage to the system itself [1]. The maintenance cost of these systems over their operational lifespan can often be much higher than the initial cost of the system [2]. Therefore, improving maintenance can significantly reduce the overall cost of the system over its operational life.

Complex mechanical systems are primarily maintained via preventive maintenance [3]. In recent decades, condition-based maintenance methods have been developed to facilitate the maintenance of the system based on its health condition [4]. These methods enhance system availability, prevent accidents, and, in some cases, reduce the need for replacing perfectly healthy components [3,5].

**Figure 1:**
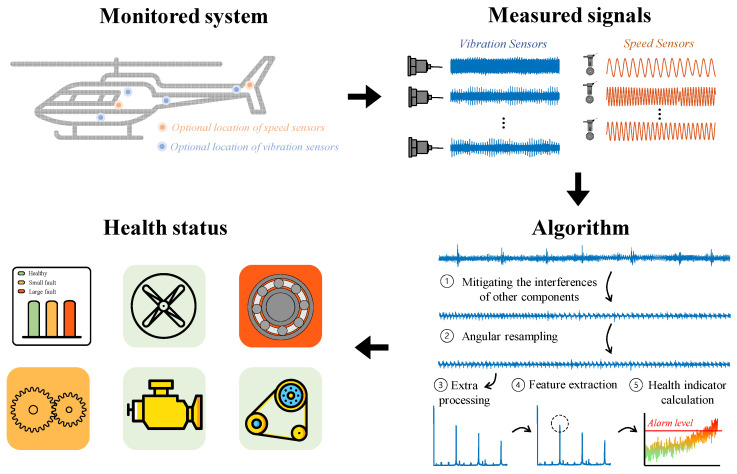
Condition-based maintenance by vibration analysis: use of vibration and speed sensors for monitoring the rotating machinery. The quantity of vibration and speed sensors is not necessarily identical.

One common approach to condition-based maintenance in complex rotating machinery is the use of vibration analysis [6]. In this approach, as depicted in Figure 1, vibration sensors are installed near the rotating components of the system (e.g., a helicopter’s rotor, gear casing, etc.), and signal processing algorithms are employed to detect faults and classify their sources [7,8]. Once the fault is detected, maintenance actions can be taken to prevent the fault from deteriorating further. The algorithms employed among different rotating components typically rely on the same fundamental methods.

A profound understanding of the component’s physical behavior guides signal-processing algorithms tailored uniquely for each component type. Most of the algorithms are based on two principles: (1) most of the information about the health status is concentrated in a finite number of frequencies due to the periodic nature of the rotation. (2) The component’s characteristic frequencies can be calculated based on its specifications, such as dimensions, component type, and so on.

There are various rotating components that can be monitored. This article focuses on fundamental methods and illustrates their application in monitoring roller bearings and gears [9,10]. The application of these principles to other components and fault types can often be achieved relatively easily. Examples of additional algorithms, monitored components, and fault types can be found in Randall’s book [11], where many of the algorithms are based on signal processing principles described in Braun’s book [12]. Jablonski’s book [13] provides numerous MATLAB implementations of these algorithms.

Section 2 provides an overview of basic fault types in rotating components and vibration measurement. In Section 3, the fundamental methods for processing vibrations of rotating components are discussed, while Section 4 demonstrates how these methods can be applied to detect and classify faults in bearings and gears. The tutorial is accompanied by videos, code, and data examples in Python and MATLAB, which are available via the link in reference [14].

## 2. General Framework: Goal, Fault Types, and Sensors

Rotating machines, such as helicopters and trains, consist of a wide variety of rotating components like bearings, gears, and shafts. For example, Sikorsky UH-60 Black Hawk has more than 100 different monitored components, including over 50 bearings, 25 gears, and 30 shafts. Throughout the life of these systems, various faults can occur, either during maintenance activities (e.g., the improper assembly of one of the components) or via gradual degradation, such as the development of a fault in the outer race of the bearing due to cyclic loading of the rolling elements on the ring [5,15].

**Figure 2:**
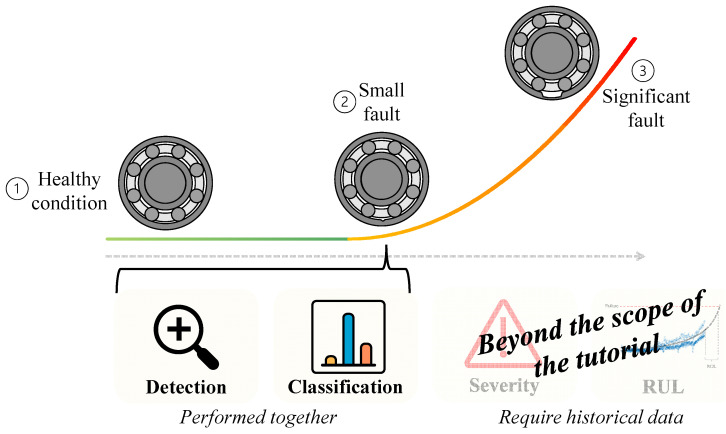
An illustration of condition-based maintenance algorithms goals: detection, classification, severity estimation, and remaining useful life (RUL) estimation.

As illustrated in Figure 2, the health condition of a rotating component can roughly be divided into three phases: (1) a healthy condition, (2) the presence of a small fault that grows slowly, and (3) a significant fault that grows rapidly. The primary goal of condition-based maintenance is to first understand if a fault has occurred in one of the rotating components of the system and, if so, which one. These two tasks are essentially performed together, meaning that the algorithm works in a way that if it detects a fault, it immediately classifies it, as will be explained in Section 4.

The two subsequent goals of condition-based maintenance are fault severity estimation and remaining useful life estimation. There are methods for achieving these goals, but they often require historical data on faults [16], making them less relevant to critical rotating systems such as helicopters. Furthermore, the algorithms for these goals are not based solely on signal processing but on statistics and machine learning. Hence, these two goals are beyond the scope of this tutorial.

**Figure 3:**
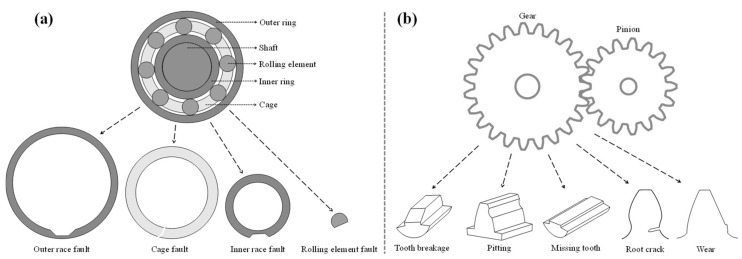
Examples of faults in rotating components: (**a**) examples of bearing faults; (**b**) examples of gear faults.

In general, each rotating component exhibits unique faults associated with it. In bearings, there are four common faults that the literature usually deals with, as shown in Figure 3a: outer race fault, cage fault, inner race fault, and rolling element fault [15,17]. Gears also have a variety of tooth faults that the literature addresses, such as illustrated in Figure 3b: tooth breakage, pitting, missing tooth, and root crack [18,19].

As presented in Figure 1, two common sensor types are used for health monitoring via vibration analysis, vibration sensors [20] and speed sensors [21], where the latter supports the analysis of the first, as will be explained in Section 3. The measured signals are analyzed using the condition-based maintenance algorithm, and the health status is estimated based on the algorithm output. The measured signal consists of the vibrations of several rotating components, random noise, and the effects of the transmission path [22].

It is worth noting that there are other sensing methods within the condition-based maintenance paradigm, like oil–debris monitoring, acoustics, temperature, and more, but they are outside the scope of this tutorial. Vibration sensors are usually preferred over others because they allow for the early detection of faults [23]. Furthermore, various machine learning techniques, often designated as artificial intelligence methods, have been applied to condition-based maintenance via vibration analysis [24,25,26,27]. For fault classification, these approaches prove highly valuable when sufficient data, including faulty data, are available. However, they are less relevant in cases where faulty data are rare [16,28].

## 3. Basic Methods and Principles

As explained in Section 1, most of the algorithms are based on two principles: (1) most of the information about the health status of rotating components is concentrated in a finite number of frequencies. (2) These frequencies can be calculated based on the specifications of the component. This insight can explain most of the stages of vibration analysis.

**Figure 4:**
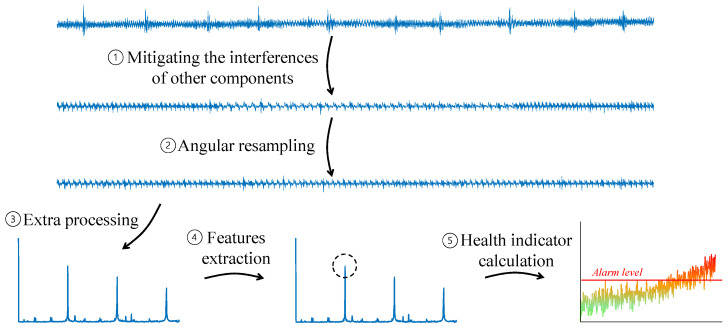
The general stages of signal processing for condition-based maintenance of a rotating component.

As illustrated in Figure 4, vibration analysis is generally utilized as follows: for each rotating component, (1) the algorithm isolates the component of interest, mitigating interferences from other sources then (2) angularly resamples the signal to be synchronized in phase, not in time, thereby ensuring a consistent number of samples in each cycle. After that, (3) additional operations are performed to improve the signal-to-noise ratio or highlight specific signal characteristics. (4) Features correlated with health status are extracted. Finally, (5) a health indicator classifies faults in the monitored component via the smart aggregation of features extracted from both the current record and healthy baseline records.

In this section, basic methods that form the basis of vibration analysis for condition-based maintenance will be described. The next section will explain how these methods can be used to detect faults in bearings and gears. All the data used in the tutorial are based on a bearing experiment with an outer race spall from the publicly available Paderborn University bearing dataset [29], a gear experiment with tooth breakage provided in references [30], a simulated gear signal generated from the dynamic model described in reference [16], and artificial white noise. The data are available via the link provided in reference [2].

### 3.1. Angular Resampling

The vibration signal is periodic relative to the phase of the rotating shaft and not to the time, as the rotational speed is never constant due to small speed fluctuations. To overcome this issue, angular resampling can be employed [31,32] as a transformation between two domains: time domain and cycle domain.

**Figure 5:**
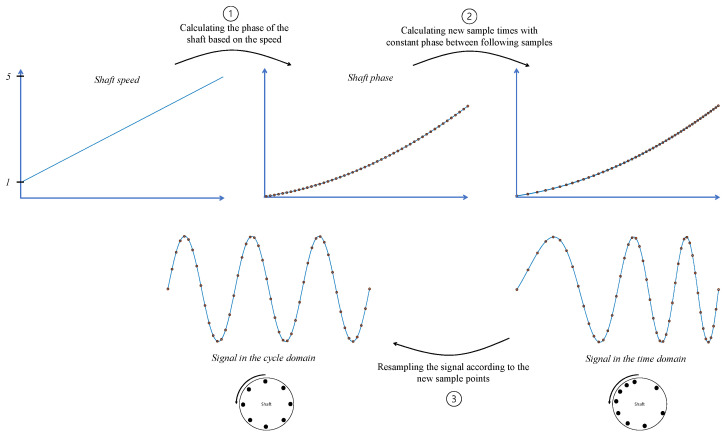
Block diagram of angular resampling.

To perform angular resampling, the phase of the shaft is calculated as a function of time, and then a new sample time vector is computed to maintain a constant phase interval between consecutive coordinates. Afterward, the vibration signal is resampled according to the new sample time vector. This process is described in Figure 5.

There are several terms in the literature to describe the new cycle domain. In this paper, we use the term “cycle domain” since the signal is synchronized to the cycle of the shaft. The frequency domain of the cycle is referred to as the “order domain”. In the context of transitioning between domains from time to cycle, the processing can be considered to have two steps: transitioning from time to cycle to make the signal truly periodical and then transitioning from cycle to order where the relevant signal information is concentrated into a finite number of specific orders.

**Figure 6:**
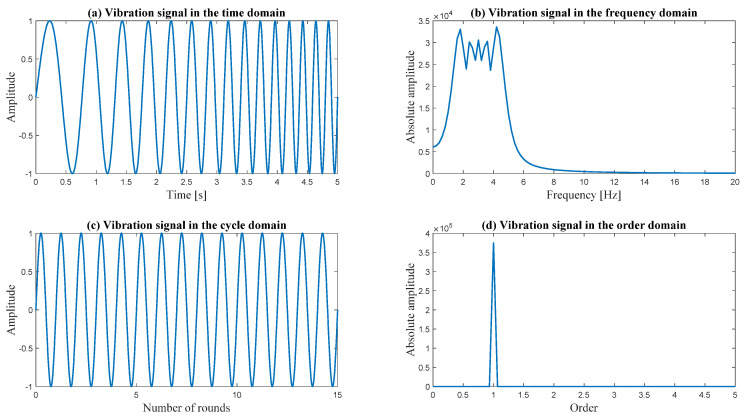
Demonstration of the advantage of angular resampling.

For example, as shown in Figure 6a, initially, the time interval between each pair of time steps is constant. The speed increases linearly from 1 Hz to 5 Hz. Consequently, the signal is smeared in the frequency domain, as can be observed in Figure 6b. As depicted in Figure 6c, in the cycle domain, the vibration is periodic, resulting in a sharp peak in the order domain, as shown in Figure 6d.

### 3.2. Synchronous Averaging

**Figure 7:**
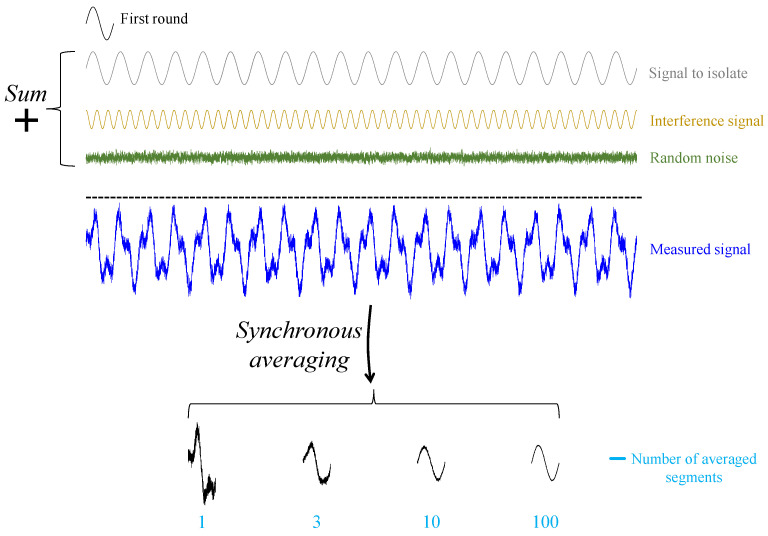
Illustration of the ability of synchronous averaging to isolate the vibrations of interest by reducing interferences and random noise.

The goal of synchronous averaging is to isolate the vibrations of the component of interest. It does so by improving the signal-to-noise ratio via reduction interferences from other rotating components [33,34] and random noise [35]. Figure 7 illustrates the ability of synchronous averaging to isolate the vibrations of interest.

**Figure 8:**
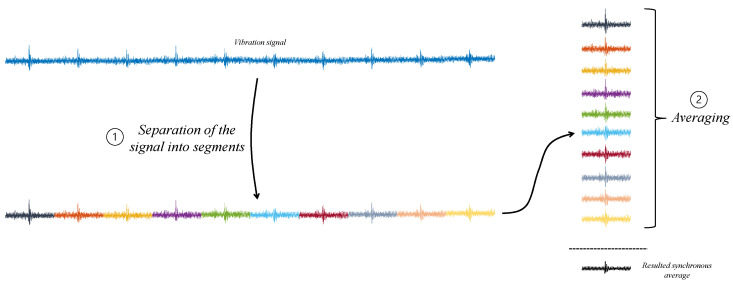
Block diagram of synchronous averaging. The colors represent different segments corresponding to complete rounds of the shaft.

In synchronous averaging, the signal is divided into consecutive segments corresponding to complete rounds of the shaft, and these segments are then averaged together [34,36]. This process is illustrated in Figure 8.

Synchronous averaging is highly sensitive to small fluctuations and is therefore not relevant to bearings, where, due to slippage, the signal in the cycle domain still exhibits fluctuations.

The reduction in random noise can be easily analyzed using Equations (1) and (2). The synchronous average is calculated using Equation (1), where sa represents the calculated synchronous average with N samples, M is the number of averaged segments, and sig is the signal in the cycle domain with M·N samples. Assuming the random noise is independently identically distributed, we can conclude that the original variance of each sample is reduced from $\sigma^{2}$ to $\frac{\sigma^{2}}{M}$, as shown in Equation (2).

(1) $$sa\left[n\right]=\frac{1}{M}\sum_{m=0}^{M-1}sig\left[m\cdot N+n\right]$$

(2) $$Var\left(sa\left[n\right]\right)=Var\left(\frac{1}{M}\sum_{m=0}^{M-1}sig\left[m\cdot N+n\right]\right)=\frac{1}{M^{2}}\sum_{m=0}^{M-1}Var\left(sig\left[m\cdot N+n\right]\right)=\frac{1}{M^{2}}\cdot M\cdot \sigma^{2}=\frac{\sigma^{2}}{M}$$

**Figure 9:**
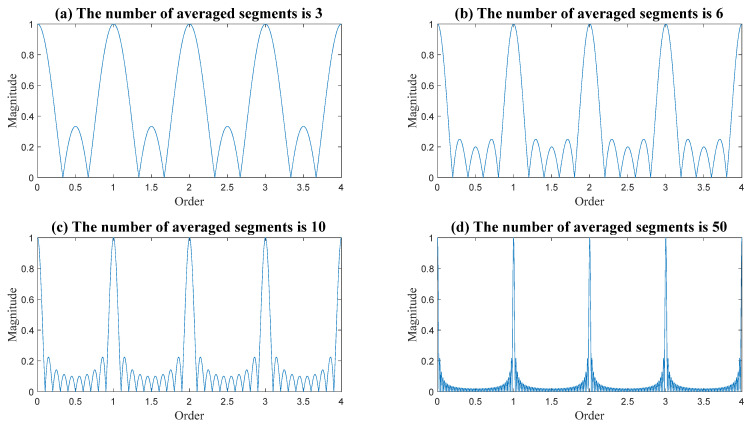
The generated filter via synchronous averaging concerning the sampled original infinite continuous signal. As the number of averaged segments increases, the filter becomes more selective with respect to the complete orders of interest.

The reduction in interference signals was analyzed in several studies, including Refs. [34,37]. Concerning the sampled infinite continuous signal, synchronous averaging creates a filter that isolates the complete orders of interest, as depicted in Figure 9. When the number of averaged segments is increased, the filter becomes more selective [38].

**Figure 10:**
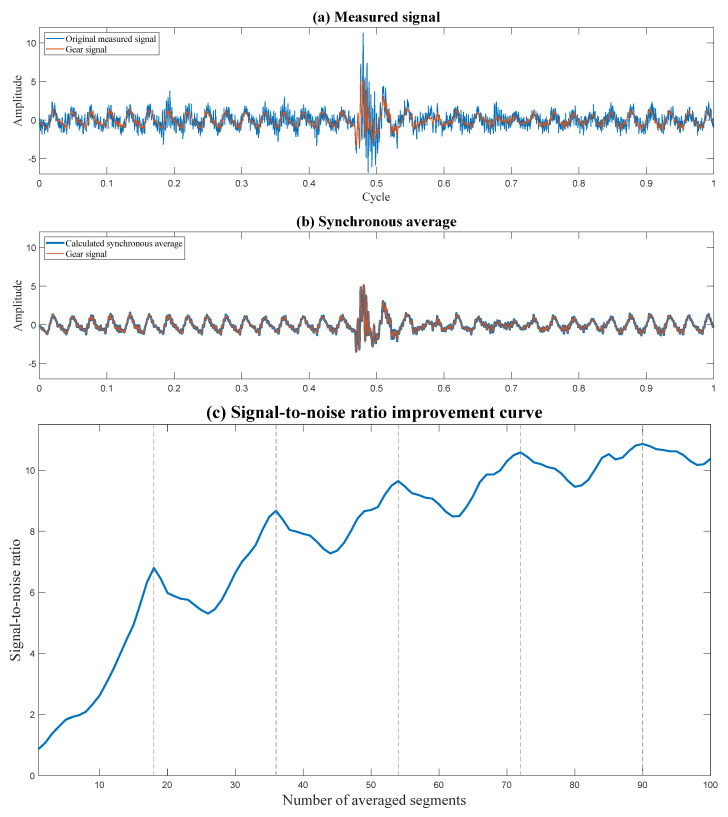
Demonstration of the effect of synchronous averaging. The number of teeth on the other wheel is 18.

Figure 10 demonstrates the effectiveness of synchronous averaging on gears. In Figure 10a, the measured signal is depicted alongside the gear signal. Due to interference from the other wheel and random noise, the measured signal differs from the gear signal. After synchronous averaging, as shown in Figure 10b, most of the interferences and random noise are reduced; thus, the synchronous average resembles the gear signal. Figure 10c illustrates the improvement in signal-to-noise ratio as a function of the number of average segments. As the number of segments increases, the effect of random noise is reduced. The number of teeth on the other wheel is 18; therefore, its interferences are eliminated when the number of segments is an integer multiple of 18. This is because the other wheel’s orders align precisely with zeros, similar to the zeros in the filters illustrated in Figure 9. This example demonstrates the possibility of choosing a number of averaged segments that completely eliminate an interfering signal [36].

**Figure 11:**
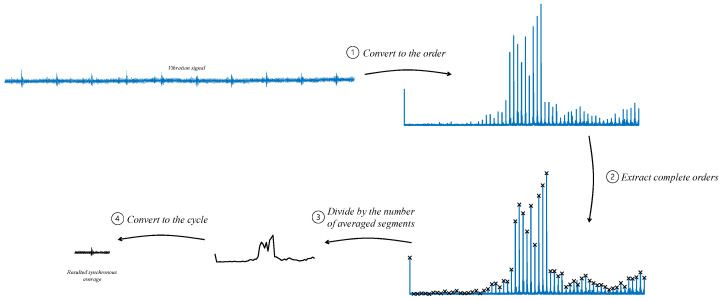
Block diagram of synchronous average calculated using the order domain by extracting orders of interest.

Regarding the statement, “most of the information about the health status is concentrated in a finite number of frequencies due to the periodic nature of the rotation”, synchronous averaging can also be calculated via the order domain by extracting orders of interest. As depicted in Figure 11, the signal is converted to the order domain, and then the values of the integer orders are extracted. After division by the number of averaged segments, the signal is converted back to the cycle domain. The resulting signal is the same synchronous average calculated using averaging in Figure 8 [2]. This analogous process demonstrates that synchronous averaging is a procedure that isolates a finite number of orders of interest of the monitored component, where most of the information about the health status is concentrated.

### 3.3. Difference Signal

**Figure 12:**
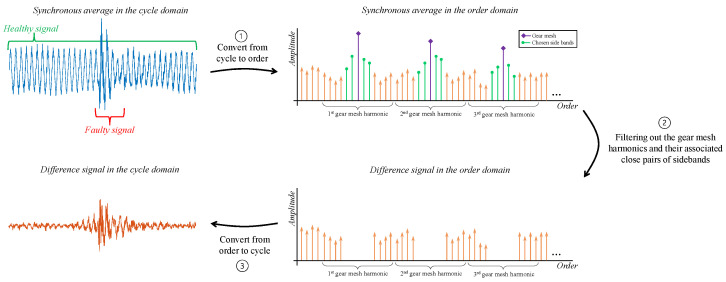
Block diagram of difference signal calculation.

Gear mesh vibrations dominate the synchronous average even under a healthy state, masking the faulty signal. This masking effect can be addressed by analyzing the difference signal [39]. The difference signal is calculated by filtering out the gear mesh harmonics and their associated close pairs of sidebands, as depicted in Figure 12 [9]. The number of filtered sidebands can be determined via trial and error; typically, two pairs surrounding the gear mesh harmonics is a reasonable value.

**Figure 13:**
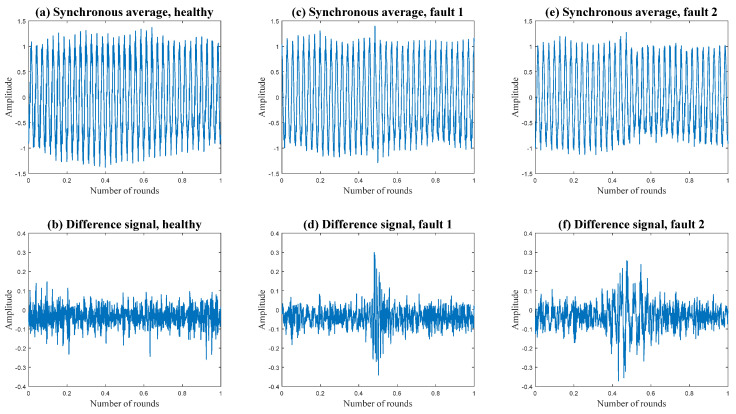
Demonstration of the effect of difference signal calculation.

In Figure 13, the effectiveness of calculating the difference signal is demonstrated. Two synchronous averages of two faulty cases are depicted in Figure 13c,d. The fault is not visible in these figures. However, after calculating the difference signal, the fault becomes clearly visible. For comparison, a healthy case is depicted alongside.

### 3.4. Dephase

For rotating components that still exhibit speed fluctuations after angular resampling, such as bearings, synchronous averaging is not effective in isolating the component vibrations. Therefore, Dephase can be employed [40,41]. Dephase can be considered the opposite of synchronous averaging. While synchronous averaging retains the signal of the synchronous components, such as shafts and gears, Dephase filters them out. There are also alternative approaches, such as employing cepstrum analysis [42,43] for “liftering” out the synchronous components [44,45].

**Figure 14:**
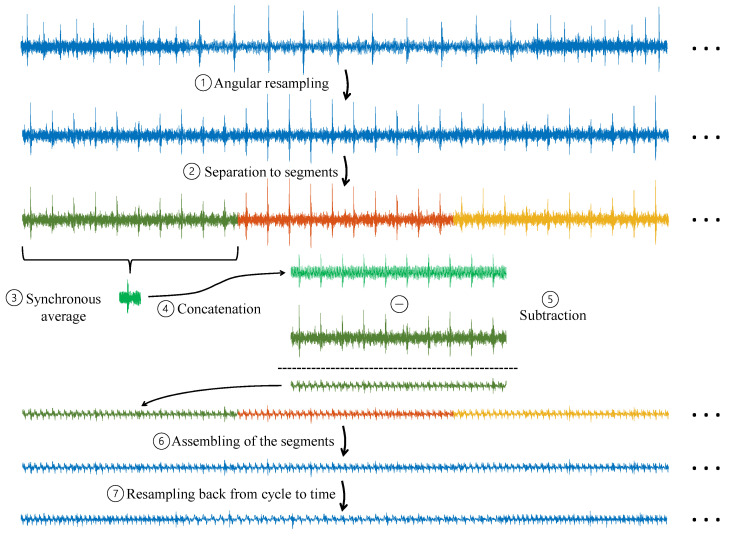
Block diagram of Dephase.

As depicted in Figure 14, for each synchronous component that needs to be filtered out, Dephase performs the following steps: (1) the signal is angularly resampled according to the desired shaft speed, and then (2) the signal in the cycle domain is divided into long segments. (3) For each long segment, the synchronous average is calculated, and then (4) concatenated to the original length of the long segment. Afterward, (5) the concatenated signal is subtracted from the original signal, and then (6) the long segments are assembled. Finally, (7) the signal is resampled back to the time domain. This process is repeated for each interfering synchronous component, and in the end, the signal of the diagnosed component remains.

**Figure 15:**
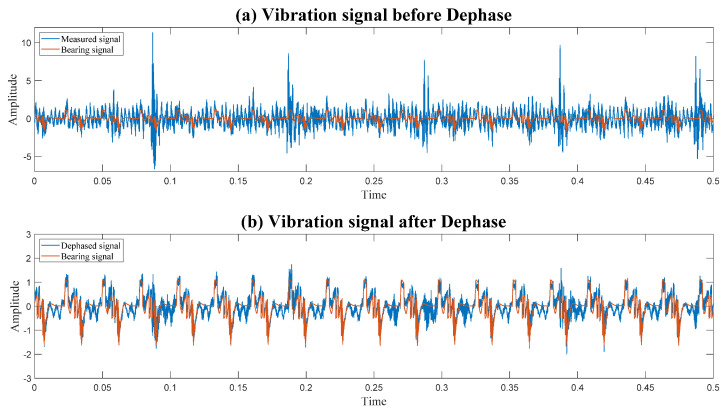
Demonstration of the effect of Dephase.

Figure 15 illustrates the ability of Dephase to mitigate the interference of gear on the vibration of the bearing. To emphasize the Dephase effect, the original signal contains a faulty gear signal.

### 3.5. Envelope Analysis and Bearing Tones

The vibration of a faulted bearing is composed of periodic disturbances [7,46]. The specific vibration shape is not as crucial for bearing diagnosis; instead, it is the frequency of the vibration that matters. Due to the high-frequency interaction signal and bearing slippage, the direct analysis of the vibration in the cycle and order domains (after angular resampling) is problematic. Therefore, the envelope of the bearing signal is analyzed [15,46].

**Figure 16:**
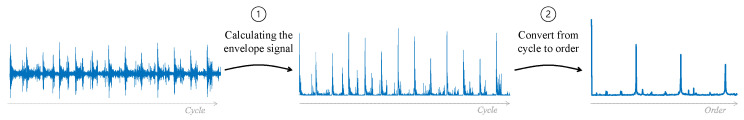
Envelope analysis of bearing vibrations.

The envelope signal of the bearing is extracted using the Hilbert transform [47,48]. Subsequently, the signal is converted to the order domain, as depicted in Figure 16.

For bearings, it is possible to calculate the relationship between the bearing parameters and the orders at which faults will manifest, referred to as bearing tones. The terms bearing tones, bearing frequencies, and bearing orders are used interchangeably.

**Figure 17:**
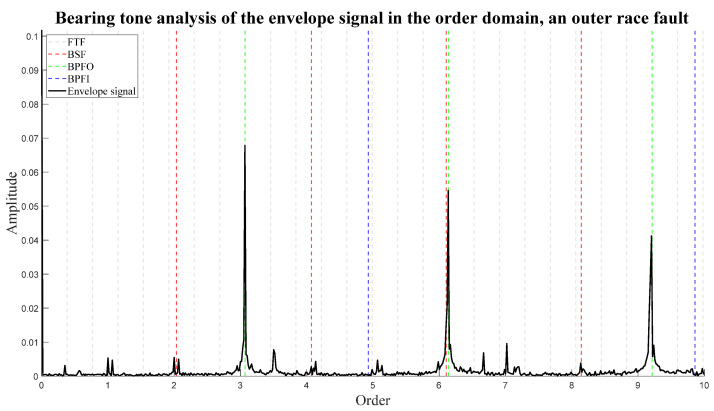
An example of bearing tone analysis of the envelope signal in the order domain. The signal is from the publicly available Paderborn University bearing dataset [29].

The bearing tones can be calculated using Equations (3)–(6) [7,15]. Figure 17 illustrates an example of these orders for an outer race fault. FTF is the fundamental train frequency, BSF is the ball-spin frequency, BPFO is the ball-pass frequency outer race, BPFI is the ball-pass frequency inner race, $f_{s}$ is the shaft speed, d is the rolling element diameter, D is the pitch diameter, α is the bearing contact angle, and n is the number of rolling elements. For analysis in the order domain, $f_{s}=1$.

(3) $$\begin{array}{c} FTF=\frac{f_{s}}{2}\left[1-\frac{d}{D}\cos\alpha \right] \end{array}$$

(4) $$\begin{array}{c} BSF=\frac{D}{2d}\left[1-{\left(\frac{d}{D}\cos\alpha \right)}^{2}\right] \end{array}$$

(5) $$\begin{array}{c} BPFO=\frac{nf_{s}}{2}\left[1-\frac{d}{D}\cos\alpha \right] \end{array}$$

(6) $$\begin{array}{c} BPFI=\frac{nf_{s}}{2}\left[1+\frac{d}{D}\cos\alpha \right] \end{array}$$

## 4. Gear and Bearing Diagnosis

In this section, we will demonstrate the diagnosis of gears and roller bearings based on the methods presented in the previous section. The two demonstrations will illustrate how to diagnose a single gear and a single roller bearing in the rotating machine. In both cases, we assume that examples of healthy records are available, either based on the initial condition of the rotating component being assumed to be healthy or on historical data from previous cases.

There are, of course, more approaches to the diagnosis of gears and roller bearings presented in various papers [15,19,49]. More examples, which will help interested readers deepen their learning on the subject, can be found in the references [15,17,50,51] for bearings and in the references [52,53,54] for gear diagnosis.

### 4.1. Gear Diagnosis

**Figure 18:**
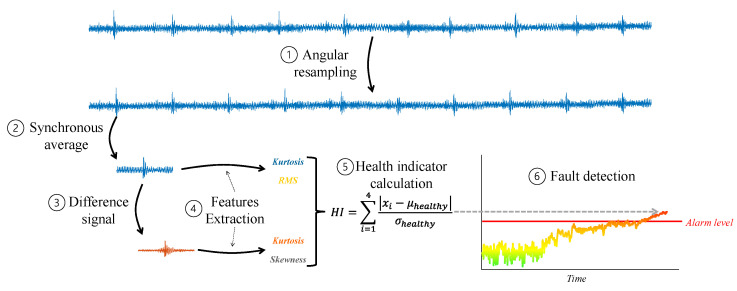
Block diagram of gear diagnosis.

As illustrated in Figure 18, initially, (1) the signal is angular resampled according to the rotational speed of the gear shaft, and then (2) the synchronous average of the gear is computed for isolating the gear vibrations. Then, (3) the difference signal is calculated from the synchronous average to highlight the defect. (4) From these signals, various statistical features can be extracted, such as root mean square (RMS), Skewness, Kurtosis, and so on. Then, (5) based on healthy data, a health indicator is calculated for the rotating component. (6) If the health indicator exceeds a certain threshold, the system alerts for a fault in the monitored component. This threshold can be determined using statistical tools or via trial and error by the operator, taking into account the trade-off between false alarms and misdetections.

It is worth noting that currently, there is no widely known technique to classify the type of fault (i.e., pitting, breakage, etc.) without the information of former gear faults in the system. Furthermore, there are also other types of processing in Step (3), apart from the difference signal and other possible features to extract in Step (4) [55]. Additionally, there are more options for health indicator calculation. Figure 18 illustrates a representative example of gear diagnosis, but of course, many other options are available.

### 4.2. Roller Bearing Diagnosis

**Figure 19:**
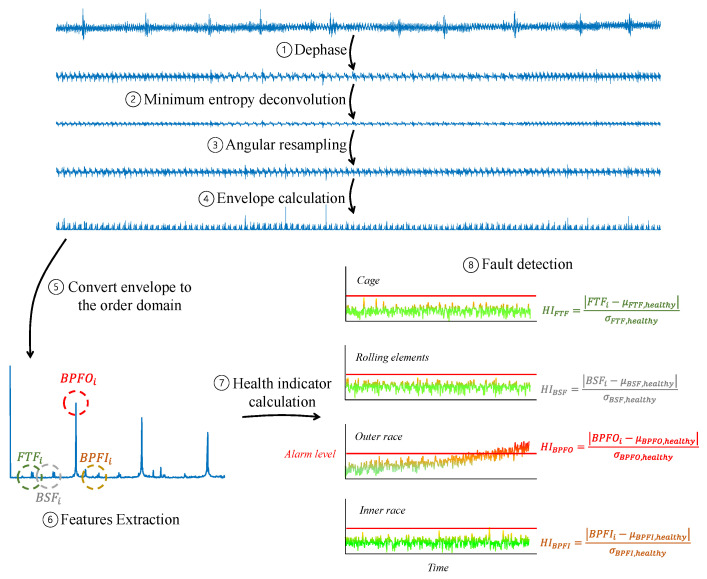
Block diagram of bearing diagnosis.

Figure 19 illustrates an optional diagnostic process of a roller bearing. Initially, (1) interferences of synchronous components such as gears are attenuated using Dephase. Then, (2) In many cases of incipient faults, signal processing techniques, such as minimum entropy deconvolution [56,57], are employed to enhance the fault signature. Following (3), the signal is angularly resampled according to the shaft’s bearing. (4) The envelope signal of the bearing is calculated, followed by (5) conversion of the envelope from the cycle to the order domain using discrete Fourier transform or power spectral density calculation. (6) Now, based on the parameters of the bearing, the bearing tones are extracted as features for fault detection. (7) Next, similar to gear diagnosis, based on health records, the health indicator for each type of fault is computed. (8) When the health indicator crosses the alarm level in any of them, the algorithm alerts about a fault in the bearing, as well as its type (cage fault/rolling element fault/outer race fault/inner race fault).

Additional examples that can assist interested readers in deepening their understanding of bearing diagnosis can be found in the references [15,17,50,51].

## 5. Summary

In this tutorial, accompanied by code and explanatory videos, we explore the fundamental methods currently used in academia and industry for the purpose of signal processing of rotating machinery for condition-based maintenance via vibration analysis. It provides a quick entry point for interested signal processing experts in the field and can also assist researchers in machine learning to become familiar with common methods used today.

As explained in the paper, the leading principle is that rotating components generate periodic signals, and therefore, most of the relevant information about their condition can be found in a finite number of frequencies that can be calculated based on the parameters of these rotating components.

Currently, fault severity and remaining useful life estimation are crucial research goals in the signal processing of rotating machinery for condition-based maintenance via vibration analysis. We anticipate that future studies will expand the existing capabilities of fault detection and classification to achieve these objectives.

## Data Availability

Code and videos of the paper are available via the following link: https://github.com/omriMatania/sp_for_cbm_of_rotating_machines_using_vibration_analysis_tutorial (accessed on 8 January 2024).
